# Creation and Acceptability of a Fragrance with a Characteristic Tawny Port Wine-Like Aroma

**DOI:** 10.3390/foods9091244

**Published:** 2020-09-06

**Authors:** Alice Vilela, Rita Ferreira, Fernando Nunes, Elisete Correia

**Affiliations:** 1 Chemistry Research Centre (CQ-VR), Department of Biology and Environment, School of Life Sciences and Environment, University of Trás-os-Montes and Alto Douro, P-5000-801 Vila Real, Portugal; 2 Enology and Viticulture Student Master, Department of Biology and Environment, University of Trás-os-Montes e Alto Douro, P-5000-801 Vila Real, Portugal; ri.230@hotmail.com; 3 Chemistry Research Centre (CQ-VR), Department of Chemistry, University of Trás-os-Montes e Alto Douro, P-5000-801 Vila Real, Portugal; fnunes@utad.pt; 4 Center for Computational and Stochastic Mathematics (CEMAT), Department of Mathematics, IST-UL, Av. Rovisco Pais 1, 1049-001 Lisboa, Portugal; ecorreia@utad.pt

**Keywords:** port wine, fragrance, benzaldehyde, sotolon, vanillin, sensory analysis, consumers acceptability, JAR scale

## Abstract

Port wine, the ultimate expression of the Demarcated Douro Region’s (DDR’s) history, cultural heritage experience, and art, was born on the slopes of the Douro river valley. One of the categories of port wine that is much appreciated by consumers is tawny port wine. This category of wine is aged in oak barrels and is characterized by oxidative aromas. Thus, the objective of the present work was to develop a tawny port wine-like fragrance, the first according to the literature. First, a group of 50 consumers in an informal environment and using two samples of tawny port wine (13 and over 40 years of aging in wood, respectively) was asked about the pleasantness of the aromas and the possible use of a tawny port wine-like fragrance. More than 80% of the group stated that they would use the fragrance as an air freshener (scent marketing in restaurants) or even in personal-use products. Then, a sensory panel of 12 participants (3 men and 9 women) was trained, and using tawny port wines of various brands and ages, the panel selected six descriptors to aromatically describe this type of wine. For the descriptors, seven aromatic chemical compounds were appointed and fragrances were developed with contributions from the panel. After several sessions with the sensory panel, three fragrances were selected, created with only three of the aromatic compounds initially used: benzaldehyde, sotolon, and vanillin. Afterward, the levels of consumer acceptability (150 individuals) for the three developed fragrances were studied and the optimization of their sensory characteristics was evaluated using a “just about right” (JAR) scale. It was found that male individuals assigned higher ratings and preferred fragrance 5.1, which was a statistically significant result (*p* < 0.001). Regarding age, Tukey’s test showed significant differences in responses to fragrance 5.3 between young adults and middle-aged adults (*p* = 0.018). Young adults gave higher scores for this fragrance. Additionally, consumers considered that the intensity of the tawny port wine aroma was ideal in the three fragrances, however, the fragrance color was not very intense. The use of the three compounds (benzaldehyde, sotolon, and vanillin) seems to be enough to obtain a tawny port wine-like fragrance.

## 1. Introduction

Sensory analysis is widely used in the food and cosmetics industries [1,2,3,4]. Sensory testing is often included for product quality assurance, as it has important advantages. Firstly, tasters have the ability to detect compounds that cannot be detected by analytical procedures and can define important sensory characteristics, identify the presence or absence of noticeable sensory differences, and of course, can decide whether or not they like a product [5].

Regardless of the industry, the primary function of sensory analysis is to run valid and reproducible tests that provide reliable data with which business decisions can be made [6]. In modern industries, sensory evaluation is a key tool in the development of new products. It allows the qualification of human perceptions related to a product, identifies the attributes that should or should not be present and at what intensity, and helps professionals to develop high-quality products that are likely to be accepted by consumers [7]. Nevertheless, in some products, the fragrance is one of the most important quality characteristics and the one that most influences product acceptance [8]. Marketing and development studies have found that fragrance can be a determining factor in the success or failure of a product. A well-crafted fragrance can provide the sensory attributes needed for a product to achieve market success and be used in sensory marketing [9,10].

The fragrance and flavor industry has focused on product evaluation through instrumental and analytical methods, such as gas or liquid chromatography, mass spectroscopy, and modern electronic noses [11]. Although the latter identify and quantify the aromatic compounds present in a fragrance, they do not provide parameters for prediction of the perception of the product by consumers; for example, they cannot judge whether the fragrance has an excessive floral note or if it will please the consumer.

In assessing the quality of fragrances and their raw materials (natural or synthetic perfume ingredients), the human sense of smell is highly selective and has an exceptional detection limit for a wide variety of compounds. This is essential since, as in perfumery, approximately 2000 to 3000 different raw materials are used, which can be divided into several groups, containing citrus, herbaceous, fruity, spice, wood, and vanilla notes. Nevertheless, perfume comprises only 15 to 30% of the fragrance in the alcohol solution [12].

Red port wine, produced in the Portuguese Demarcated Douro Region (DDR), is divided into several categories according to the type of aging. Tawny port wines are aged by oxidation in 500 L wooden barrels. Tawny port wines with an age indication are exceptionally high-quality port wines. The permitted age indications are 10, 20, 30, and over 40 years old. As a rule, a 10-year-old tawny port is a blend of wines whose characteristics match a wine of that age. The evolution of these wines over time is evident from the aromas of dried fruits, toasted vanilla, and oak notes, and in the range of colors that evolve, from reddish-amber to golden [13]. In wines aged for 30 and 40 years, the characteristics are more concentrated and complex, containing notes of honey and spices, together with deep aromas of apricots, dried figs, and hazelnut [13,14].

To enhance and keep one of the richest products in the Douro region alive, the objective of this work was to develop a tawny port wine fragrance—the first according to the literature. This fragrance could be used as an air freshener in gourmet restaurants. The restaurant industry is increasingly competitive, and in the fight to rise above the rest, “out-of-the-box” ideas are necessary. Marketing efforts have extended to the olfactory system, since scent is becoming a strategic tool for restaurants who want to improve brand recognition and sales. Additionally, the appeal of smell over visual or audible marketing is undetected by most customers, but is highly effective in creating a food-friendly mood. Moreover, fragrances could also be used in enogastronomy in elegant dining, in which chefs use foams and smokes to enhance the dining experience, or could even be used as a flavoring ingredient in some gourmet dishes, ranging from starters to main courses and desserts.

In the first phase, the possible acceptance by consumers of a fragrance with these aromatic characteristics was assessed. Afterward, a panel of tasters was trained to select the most significant aromatic descriptors for tawny port wines. After fragrance development, their acceptance by the consumers was evaluated using a “just-about-right” (JAR) scale. JAR scaling combines the measurements of attribute intensity and consumer acceptability [15]. This scale is commonly applied in the food industry for product development [16] and is used in the early stages of product elaboration [17,18], mainly when a systematic solution, like the full design formulation, is not available. This technique is also used in marketing and R&D departments due to its ease of use and directional guidance [19]. Moreover, and according to Lawless and Heymann [20], JAR scales are an easy way to determine the optimal level of an attribute’s intensity.

## 2. Materials and Methods

### 2.1. Acceptancy and Usability of a Tawny Port Wine-Like Fragrance by Consumers

To verify the acceptancy and usability, by consumers, of a fragrance characteristic of tawny port wine, a hedonic sensory test was carried out by 50 individuals, in which the olfactory perception of two different tawny port wines of different ages were evaluated—a new, 13-year-old tawny port and an older one with over 40 years old (40+). In this way, we had a relatively new wine and an incredibly old one. The perceived aromas are different, due to the aging phenomenon, but the core is identical, with nutty characteristics, coffee, dried fruits, toasted vanilla, and oak notes, among others. In this way, with only two samples, we covered the aromas from a “simple” tawny to a “more complex” one.

Consumers were asked about their gender, age, and main occupancy, as well as if they were port wine consumers. After smelling the tawny port wine samples, presented in aliquots of 1.5 mL and only opened when provided to the taster (consumer), they were queried about the pleasantness of its aromas and the possible use of a tawny port wine-like fragrance. Issues like: “Would you buy a product (other than port wine) where this aroma was present? Would you use the aroma in your home? Where would you use it?” were questioned.

### 2.2. Selection and Training of a Tasting Panel

For the execution of this work, a population of individuals was recruited, made up of employees from the University of Trás-os-Montes and Alto Douro (UTAD), belonging to various areas of the University. The final 25 candidates were untrained individuals who applied voluntarily, using a questionnaire (Supplementary Form 1S). This, which had the purpose of characterizing them, consisted of collecting basic information such as gender, age, and profession. Additionally, some personal questions were included, such as what their interests and motivations were; what was their availability, knowledge, and skills; existence of chronic diseases and food allergies; and food consumption habits. With this information, 12 members were selected for the panel—3 men and 9 women—aged between 45 and 60 years old.

**Figure 1:**
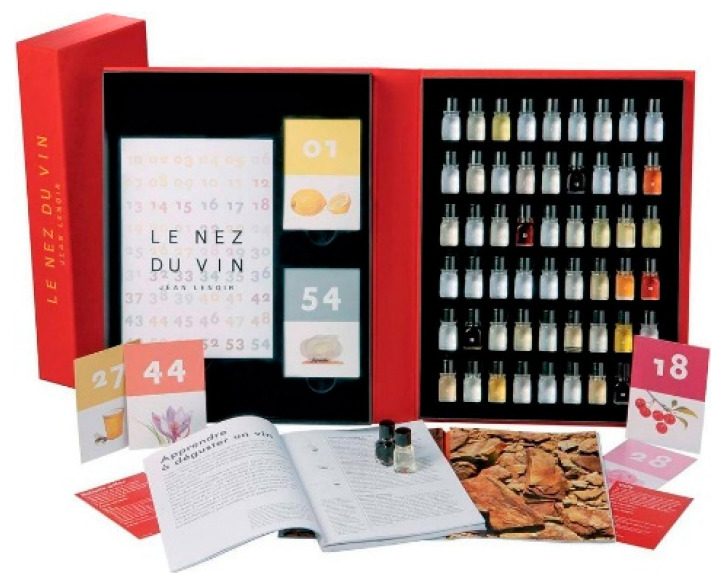
Le Nez by Vin aroma kit^®^ by Jean Lenoir. A complete perfume box to learn how to recognize odors and train the sense of smell. This set consists of 54 bottles with wine aromas.

After this first selection, candidates were familiarized with some basic concepts of sensory analysis. Then, for olfactory perception training, 3 training sessions were held with Jean Lenoir’s 54 aromas master kit “Le Nez du Vin” (Figure 1). The aromas were presented individually, and panelists were asked to write down the aroma they could identify. Sensory training took place in individual tasting booths in a sensory evaluation laboratory [21]. All evaluations were conducted from 15:00 to 17:00 h. Sessions were carried out under controlled conditions of temperature (20 ± 2 °C) and relative humidity (60 ± 20%).

### 2.3. Identification and Selection of Aromatic Descriptors of Tawny Port Wine and Corresponding Aromatic Compounds

**Figure 2:**
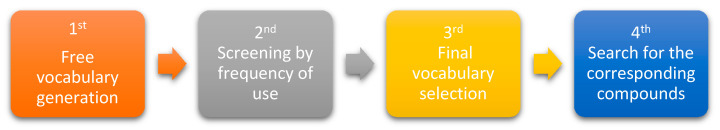
Schematic representation of the methodology used in the identification and selection of aromatic descriptors of tawny port wine.

After training the sensory panel, the ISO 11035 [22] method was used for the identification and selection of aromatic descriptors of tawny port wine. To do this, 4 steps, described in Figure 2, were followed.

In the free vocabulary generation step, a proof sheet (Supplementary Form 2S) was provided to each member of the panel. The objective was to identify the aromas/sensations that the sample of tawny port wine suggested, through vocabulary that was familiar to them. The port wine samples were placed in tasting glasses [23] and 30 mL samples of each wine were served and presented to all panel members, after explaining the purpose. This tasting took place 4 times (4 tasting sessions), on different dates, with the following wines: the first session was with Graham’s tawny (30 years old); the second with Porto Cruz tawny A (undetermined age); the third with Porto Cruz tawny B (undetermined age); and the fourth session with Warre’s Great 10-year-old tawny.

After collecting the aromatic descriptors of all sample wines, they were sorted. The descriptors of the same families were grouped, and their frequency of use was observed. In this way, the final vocabulary selection was made for the next research stage.

The ScienceDirect platform (https://www.elsevier.com/research-platforms) and the National Center for Biotechnology (https://www.ncbi.nlm.nih.gov) were used to search the literature on the various aromatic compounds of port wine. The descriptors that were listed by the panelists at a high and intermediate frequency were considered. There were descriptors whose compounds, although not identified in port wine, were described as part of the aroma of wine in general and these too have been considered. In total, 7 aromatic compounds (Table 1) were identified for the development of tawny port wine fragrances.

### 2.4. Development of Fragrances

For the development of fragrances, “mother solutions” were prepared with 10 mL of 96% ethyl alcohol (AGA—Álcool e Géneros Alimentares, S.A.) and added to seven 50 mL volumetric flasks. Approximately 0.300 g of each compound was weighed in its respective flask and made up to volume with 96% ethyl alcohol. These mother solutions were then diluted 100 times in ethyl alcohol to new 50 mL volumetric flasks, with a concentration of 600 mg/mL.

**Figure 3:**
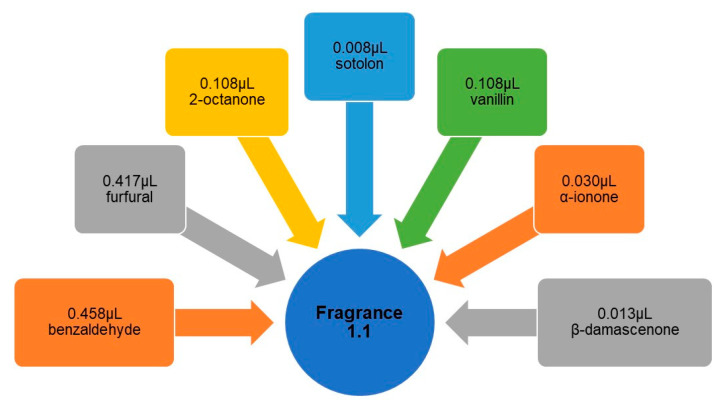
Aromatic compounds and respective quantities added to the fragrance 1.1.

For this initial development of fragrances, the thresholds of perception of each compound were considered. For the sample of fragrance 1.1, in a 50 mL volumetric flask, the quantities described in Figure 3 were added and the volume was made up with 96% ethyl alcohol (AGA—Álcool e Géneros Alimentares, S.A.). Thus, fragrance 1.1 would have all compounds with concentrations equal to their respective perception thresholds.

For the first fragrance tasting session, five more fragrance samples, from 1.2 to 1.6, were developed according to the concentrations shown in Table 2.

After the development of the 6 sample fragrances (1.1–1.6), the first fragrance tasting session with the panelists was prepared. To do this, the 50 mL flasks of all samples were shaken slightly and a little of each was poured onto their respective cardboard strips (kindly provided by Perfumes & Companhia S.A.); then, each strip was kept in a sterilized 80 mm diameter plastic Petri dish. The Petri dishes were encoded with 3-digit alphabetical codes (ABC, and its 6 variations). Petri dishes with the respective strips of cardboard soaked with fragrances were prepared 10 min before the start of the tasting. The samples were then randomly arranged on the bench of the sensory analysis laboratory, each with the respective proof sheet. The panelists were informed that they should open one Petri dish at a time, smell the fragrances, and fill out the respective proof sheets (Supplementary Form 3S).

For the development of fragrances for the second tasting session, the concentrations described in Table 2 (2nd tasting session) were used. The aromatic compounds Sotolon, β-damascenone, and α-ionone are the ones that present the smallest detection threshold. The values, according to the referenced literature, can be seen in Table 1. Therefore, we aimed to determine if the absence of these compounds in the fragrance was noticeable. With that purpose, in the second formulation (fragrances for the second tasting session), only 3 samples of test 1 with the highest values of global appreciation were made. For these samples, in a 50 mL volumetric flask, the corresponding quantities were added, and the volume was made up with a previously prepared hydroalcoholic solution. For this last solution, in a 250 mL volumetric flask, 125 mL of distilled water was added and the volume was made up with 96% ethyl alcohol (AGA—Álcool e Géneros Alimentares, S.A.). In the literature, it is described that the alcoholic fraction of a perfume can be based on a hydroalcoholic solution of 950 mL of ethyl alcohol and 50 mL of water [12]. However, we diluted the alcohol content by 50% because, according to the sensory panel comments, the intensity of the smell of ethyl alcohol was extremely high and “pungent”.

**Figure 4:**
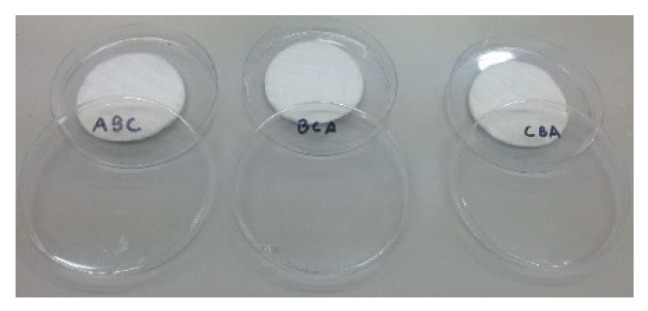
Samples of the fragrances developed for the 2nd tasting session, in the respective Petri dishes.

After developing the 3 fragrance samples, they were prepared for the second tasting session. To do this, 50 mL balloons of all samples were shaken slightly and a little of each was poured onto the respective cotton disks (MyLabel), as shown in Figure 3. Cotton disks were used instead of the cardboard strips, since it was found that the strips did not retain the sample odor for long enough. Then, each disk was stored in a plastic sterilized Petri dish of 80 mm in diameter, previously coded with 3 alphabetical digits, ABC, and 2 more variations (Figure 4). Petri dishes with the respective cotton disks soaked with fragrances were prepared 10 min before the start of the tasting.

The samples were then randomly arranged on the bench of the sensory analysis laboratory, each with the tasting sheet presented in Supplementary Form 4S. It was explained to the panelists that they should open one Petri dish at a time, smell the fragrances without touching the cotton disks, and fill out the respective sheets.

For the development of fragrances for the third fragrance tasting session, the concentrations described in Table 2 (3rd tasting session) were employed. For samples 3.1, 3.2, and 3.3, the corresponding quantities were added in a 50 mL volumetric flask and made up with 96% ethyl alcohol (AGA—Álcool e Géneros Alimentares, SA).

The 50 mL flask of sample 3.4 was made with the alcohol obtained from the distillation of a 10-year-old tawny port wine. To obtain the distillate, an automatic D.E. 2000 (Laboratoires Dujardin-Salleron) was used.

After the development of the 4 fragrance samples (3.1–3.4), they were prepared for session number three, applying the same procedure described in the second session and the form presented in Supplementary Form 5S.

For the development of fragrances for the fourth session, the concentrations described in Table 2 (4th tasting session) were used. For samples 4.1 and 4.2, the corresponding quantities were added to a 50 mL volumetric flask and made up with tawny port wine distillate obtained at the previous point (2.4.3). For sample 4.3, the concentrations present in the stock solutions of each compound were used. Thus, in a 50 mL volumetric flask, 3 mL of the stock solution of benzaldehyde, 2.90 mL of the stock solution of furfural, 0.6 mL of the stock solution of vanillin, 0.59 mL of the stock solution of 2-octanone, 0.17 mL of the stock solution of α-ionone, 0.083 mL of the stock solution of β-damascenone, and 0.054 mL of stock solution of sotolon were added and then, the volume was adjusted with the distillate prepared in 2.4.3.

After the development of the 3 fragrance samples, they were prepared for session number four, applying the same procedure described above, in the second session and the form presented in Supplementary Form 5S.

For the fifth session, 3 fragrances were developed, all using the mother solutions of each compound. For sample 5.1, 2 mL of the benzaldehyde stock solution, 0.5 mL of the vanillin stock solution, and 1.5 mL of the sotolon stock solution were added to a 50 mL volumetric flask and the volume was made up with the alcohol obtained from the distillation of a tawny port wine. For sample 5.2, 1.4 mL of the benzaldehyde stock solution, 0.8 mL of the vanillin stock solution, 1.5 mL of the sotolon stock solution, 1.49 mL of the furfural stock solution, and 0.95 mL of the β-damascenone stock solution were added in a 50 mL volumetric flask, and the volume was made up with the distillate. For sample 5.3, 1.4 mL of the benzaldehyde stock solution, 0.8 mL of the vanillin stock solution, and 1.5 mL of the sotolon stock solution were added to a 50 mL volumetric flask and the final volume was made up with the distillate. The concentrations of each compound present in these 3 samples are shown in Table 3.

After the development of the 3 fragrance samples, they were prepared for session number five, applying the same procedure previously described and the form presented in Supplementary Form 5S. All tests took place from 14:30 to 16:00 h, from April to June, in a laboratory properly equipped for sensory analysis, according to the standards of ISO 8589 [21].

### 2.5. Evaluation of Consumers’ Acceptability of the Developed Fragrances through a JAR Scale

To verify the acceptability by consumers of a characteristic tawny port wine-like fragrance, the fragrances previously developed (5.1, 5.2, and 5.3) were sensorially evaluated. The study was carried out in different locations in the north of the country (Portugal) with the participation of 150 consumers using a tasting sheet provided in Supplementary Form 6S.

### 2.6. Data Analysis

Descriptive analysis of data was presented as mean (M) and standard deviation (SD). Skewness and kurtosis coefficients were computed for univariate normality analysis purposes, and all values were within ±2. To evaluate if gender and the fact of one being a port wine consumer had a statistically significant effect on the classification of fragrances, a multivariate analysis of variance (MANOVA) was performed followed by a one-way analysis of variance (ANOVA). Box’s M test was used to test the assumption of homogeneity of variance–covariance matrices [31]. The chi-square test of independence was used to determine whether there was an association between sociodemographic variables and favorite fragrance. Cramer’s V was reported as a measure of the effect size regarding the chi-square test. All statistical analysis was performed using software SPSS 25.0 (IBM SPSS 25.0, Chicago, IL, USA). Statistically significant effects were assumed for *p* < 0.05.

## 3. Results and Discussion

### 3.1. Acceptancy and Usability of a Tawny Port Wine-Like Fragrance by Consumers

Aiming to know the acceptancy and usability of a tawny port wine-like fragrance by consumers, a preliminary screening was made. In the initial consumer’s test, a sample of 50 individuals (22 male and 28 female), randomly recruited, participated in the questionnaire. The mean age of the consumers sample was 35.76 (SD = 15.11) years, with an age range from 17 to 64. A total of 30 (60%) individuals reported being port wine consumers. Regarding the knowledge of port wine categories, 24 (48%) reported being knowledgeable. Of the interviewed individuals, 41 (82%) stated that they would buy the port wine fragrance. A total of 38 (76%) individuals stated that they would use a fragrance of port wine and 23 (56.1%) would preferably use it as an air freshener. These initial results were important for us to begin the work of preparing a tawny port wine-like fragrance, and the first step was to recruit tasters/panelists to form a trained panel.

### 3.2. Selection and Training of a Tasting Panel

Internal recruitment, at the University of Trás-os-Montes and Alto Douro (UTAD), was the methodology chosen. Out of a total of 25 candidates, 12 were selected, which is a reasonable number since a descriptive analysis panel must consist of at least five elements [20]. This selection was made, mainly, according to the person’s availability, interest, and motivation, and, finally, wine being included as part of their eating habits. By conducting internal recruitment, it was possible to have the members of the panel in one place, without the need for remuneration and with confidentiality of the results and stability over time [32]. Moreover, only a lack of availability of the tasters would be a disadvantage and a major concern.

Considering the advice in ISO 8586-1 [33], after recruitment, training was carried out with the panel members to familiarize them with some basic concepts of sensory analysis and the role of the senses; the master kit “Le Nez du Vin” by Jean Lenoir was used. The kit consists of 54 liquid aromas, typical of white and red wine, which help to develop the sense of smell and the ability to recognize and describe the aromas present in wine. In this way, the sensory acuity of each taster was trained while they had contact with products like those with which they would later work. Although the aromas are not typical of port wine or tawny, during training, we insisted on the aromas most likely to be found in these wines: almond, plum, walnut, honey, black pepper, vanilla, cinnamon, pepper, almonds baked goods, roasted hazelnut, caramel, coffee, and dark chocolate. All the tasters had included wine in their eating habits and this prior knowledge of the product to be analyzed could also be advantageous. The training was completed after 3 sessions when the tasters were able to identify at least 60% of the aromatic compounds in the master kit.

### 3.3. Identification and Selection of Aromatic Descriptors of Tawny Port Wine and Corresponding Aromatic Compounds

The ISO 11035 [22] method was used to identify and select descriptors that would establish the aromatic profile of tawny port wine. Four different tawnies were used (Graham’s tawny (30 years old); Porto Cruz tawny A (undetermined age); Porto Cruz tawny B (undetermined age); and Warre’s Great 10-year-old tawny) to have the maximum possible variability in aromatic descriptors. The most evident aromas should be the first to appear on the list, thus, the tasters started by indicating the descriptors with the highest perceived aroma intensity. Whenever possible, all descriptors were grouped into broader families, as shown in the following table (Table 4).

According to ISO 11035 [22], depending on the objectives, the most frequently used and intermediate frequency descriptors should be selected. For this reason, aromatic descriptors that showed a residual frequency were ignored, while the most frequent ones were considered. Thus, the family descriptors selected were alcohol, dry fruits, spices, wood, sweet/honey, and floral/dried flowers. After bibliographic research, the aromatic compounds that correspond to the previously selected descriptors, or descriptor families, were compiled (Table 4).

Nevertheless, it was not obvious the attribution of a descriptor to an aromatic compound already described in the literature. For example, a wine’s floral aroma descriptor easily reports to β-damascenone and/or α-ionone, primary aromatic compounds; effectively, the training carried out with the panel of tasters using the master kit of 54 aromas included these floral aromas. It is known that β-damascenone precursors can be hydrolyzed, under acidic conditions, during wine aging, leading to an increase in the concentration of β-damascenone [42]. However, it is also described that, during aging, β-damascenone can be oxidized to hydroxy-β-damascenone, an odorless compound [43]. Thus, β-damascenone contribution to the aroma of a tawny port wine may be small and the choice of this floral compound may be ambiguous. Besides, the interaction of this compound with others in the wine can give rise to a different aroma when compared to its aroma alone [44]. This could explain the fact that some panelists describe an aroma of dried flowers instead of simply floral when training with the master kit.

The question of the existence of several descriptors for a compound was also problematic. The perception threshold for sotolon is in the range of 1–100 µg/L in wine [27] and its descriptors range from wood, burnt, dried fruit, curry, nut, spices, and spicy [41,45]. Since sotolon concentrations are between 5 and 958 µg/L for wines between 1 and 60 years of age [41], the difference in the descriptors may be related to the concentration at which it is found. Thus, in small quantities, sotolon can be described by its spicy aroma [46], despite having been selected, essentially, for the descriptor wood. Therefore, sotolon contribution to the final fragrance aroma will be more complex, since it can be misleading.

### 3.4. Development of Fragrances

**Figure 5:**
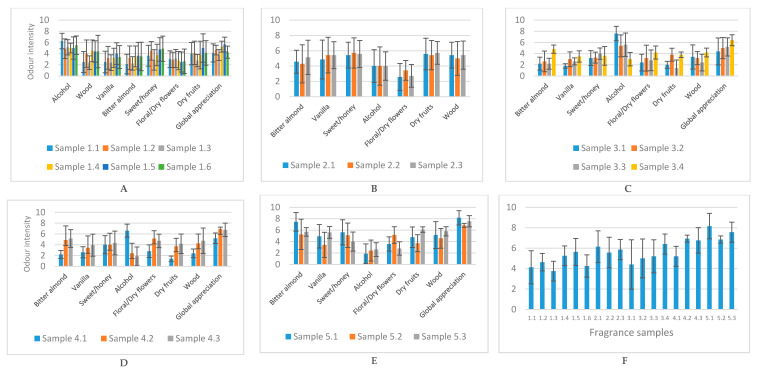
(**A**)—Mean intensity of descriptors obtained after the first sensory test with the first six fragrances developed; (**B**)—Mean intensity of descriptors obtained after the second sensory test with the three fragrances developed; (**C**)—Mean intensity of descriptors obtained after the third sensory test with the four fragrances developed; (**D**)—Mean intensity of descriptors obtained after the fourth sensory test with the three fragrances developed; (**E**)—Mean intensity of descriptors obtained after the fifth sensory test with the three fragrances developed; (**F**)—Global appreciation of all tawny port wine-like fragrances developed.

The development of the fragrances started with 7 of the 12 aromatic compounds selected (benzaldehyde, furfural, 2-octanone, sotolon, vanillin, α-ionone, and β-damascenone), those available at the beginning of this work. In this first phase, the objective was to obtain a fragrance, like tawny port wine, with identical values of intensity for all aromatic descriptors. Figure 5A shows the average global intensity (scale 1–9) of the various aromatic descriptors from tasting 1, obtained by the panelists.

During test 1, the testers’ fatigue was detected, both in terms of motivation, which decreased with the progress of the six samples, as well as due to concentration and attention. Although the number of fragrance samples was not as high (six samples), the rest between them was a maximum time of 5 min, which, according to Chambers [47], may not be enough; once, he recommended a rest of 10 min. Furthermore, concentration may have been affected by the fact that the panelists opened the Petri dishes and removed the cardboard strips to better evaluate the aroma. This generated two complications: possible contamination between samples, as well as possible external aromas and, also, the fact that the cardboard strips dry easily.

It was found that the aromatic descriptor, alcohol, was the one with the highest average intensity in practically all samples. Since the concentrations present in these samples were, at most, twice the thresholds of perception of the compounds, it would be expected that the intensity of the aromas would be minimal. This fact, combined with the use of 96% ethyl alcohol to make up the six samples, may justify the intensity of its aroma, which overlapped with all the others.

In the literature, it is described that the alcoholic fraction of a perfume can be based on a hydroalcoholic solution of 950 mL ethyl alcohol and 50 mL of water [12]. To reduce the intensity of the alcohol aroma of the samples, the use of 96% ethyl alcohol was replaced. Progress was made towards the development of new fragrances, which were made with a hydroalcoholic solution of 50% ethyl alcohol and 50% water. This solution was chosen because, and according to the panelists, the intensity of the alcohol smell was very intense. Thus, we moved on to the second test, considering the three samples of test 1 with the highest values of global appreciation (Figure 5A).

For this second test, the method of presenting the samples changed; instead of the cardboard strips, cotton disks embedded in the fragrance were used inside the Petri dishes. The panelists were asked not to touch the cotton pad and, therefore, the aroma remained for a longer time and there were no external contaminations. The evidence proof sheet was also changed; the aromatic descriptors were now in alphabetical order, instead of starting with the descriptors alcohol and wood (Supplementary Table S4). In this way, we tried to avoid the possible tendency of the panel to assume that the most intense descriptors came first.

During this second test and with only three samples, the fatigue of the tasters was not so evident. The results of this test are shown in Figure 5B. It should be noted that the aromatic intensity of alcohol was lower in these samples, compared to test 1. However, it was found that fragrances evaporated very quickly, and it was necessary to constantly return the samples to their cotton pads.

Fragrance evaporation from the cotton pads may have occurred because the aromatic compounds used are poorly soluble in water. Besides, the percentage of distilled water used in the hydroalcoholic solution was 50%. This value is relatively high compared to that described in the literature, where the authors use a percentage of distilled water between 5–25% [12,42]. Despite the decrease in the intensity of the alcohol aroma (Figure 5B), the high volatility of the remaining compounds was not advantageous. Thus, for the next test, it was again chosen to use 96% ethyl alcohol and the concentration of aromatic compounds in each sample was doubled.

Alternatively, some authors report the use of cereal alcohol or partially denatured alcohol with benzalkonium chloride for the development of fragrance products [48]. Therefore, in order not to distance from the aim of this work, alcohol obtained by the distillation of a tawny port wine was used as an alcoholic base in one of the samples. Wine spirit or “aguardente” contains high levels of higher alcohols, esters, and aldehydes, which benefits its aroma [49,50]. Therefore, we would expect that the distilled tawny port wine would also contribute to the aromatic complexity of the sample while reducing the aroma of the ethyl alcohol. The results of this third test, which includes the sample made with the distillate (sample 3.4), are shown in Figure 5C.

The difference between sample 3.4 and the others for the alcohol descriptor was notable. The use of alcohol from the distillation of a tawny port wine as the alcoholic base of sample 3.4 resulted in the reduction in the intensity of alcohol aroma. In addition, regarding the global assessment, where the panelists expressed their liking on a 1–9 scale, this sample obtained the highest value to date (Figure 5C). It should also be noted that the global intensity for all descriptors, apart from alcohol, was higher for sample 3.4 compared to sample 3.1. For this reason, the distillate seemed to enhance the aroma of the various compounds, perhaps by decreasing their volatility. After these results, the fragrances developed later all had the alcohol from the distillation of a tawny port wine as the alcoholic base.

Although factors such as volatility, retention, and polarity can be determined for each compound of a fragrance, there is no doubt that when these compounds are present in the same mixture, the interactions between them modify evaporation behavior [48]. Subsequently, it was decided that, after these results, the fragrances developed later should all have the alcohol from the distillation of a tawny port wine as the alcoholic base.

For the fourth test, it was proposed to increase the concentration of the compounds to 10 times their threshold of perception. Experimentally, a fragrance was also developed with the concentrations of the mother solutions of each aromatic compound, for a more accentuated aroma. This sample, 4.3, obtained a value close to 7 in the global assessment, together with sample 4.2 (Figure 5D). For sample 4.3, it was possible to verify an agreement in the intensities of the various descriptors. These range from 4–5, except for spices and alcohol, with these results meeting the intended objective.

The panelists were pleasantly surprised by samples 4.2 and 4.3. To take advantage of this positive feedback, panel members were asked to mark on the tasting sheet the sample that most closely resembled the aroma of a tawny port wine. It was possible to conclude that, although the global assessment was higher for sample 4.2, 67% of the panel members thought that it was sample 4.3 that most resembled the tawny port wine’s aroma. It was beneficial to be aware of this result because it was in the general interest of this work to develop a fragrance that not only resembled the aroma of tawny port wine but was also well accepted by possible consumers.

Even though sample 4.3 presented a slightly lower overall appreciation than sample 4.2, it was decided to give more relevance to the fact that this fragrance was more like the aroma of tawny port wine. Thus, new fragrances were developed with the mother solutions of each compound, like sample 4.3. To balance the concentration of compounds whose aromatic intensity was higher, namely floral/dry flowers and wood, it was decided not to include α-ionone and 2-octanone in sample 5.2. Two more samples were developed with only three compounds—benzaldehyde, sotolon, and vanillin—to understand if there would be a lack of other aromatic compounds. Interestingly, it was found that for these two samples, 5.1 and 5.3, the panel detected floral aromas even in the absence of the compounds that originate this descriptor (Figure 5E). This may be due to the chemical interactions that exist between the compounds and the mixture where they are inserted, which can alter the human perception of aroma [51]. These observations are in line with a review article written by Chambers and Koppel in 2013 [52], which shows the various scents that are associated with the compound 3-methyl-butanol. This compound has been identified in several wines and is associated with green, herbaceous, sweet, dark chocolate, varnish, and cheese aromas. In conclusion, there may not be a definitive association between a compound and an aroma. On the contrary, this association changes according to the matrix and the composition of the solution where the compound is inserted [52]. 

### 3.5. Evaluation of Consumers Acceptability of the Developed Fragrances through a JAR Scale

Aiming to verify the acceptability by consumers of a characteristic tawny port wine-like fragrance, three of the fragrances previously developed, the most appreciated by the trained panelists (5.1, 5.2, and 5.3), were sensorially evaluated by 150 consumers. The study was carried out in different locations in the north of the country (Portugal).

#### 3.5.1. Participants

A sample of 150 individuals (73 women and 77 men) randomly recruited from the north region of Portugal participated in the study. A total of 62 (41.3%) individuals reported living in a rural area, while 88 (58.7%) reported living in urban areas. The mean age of the women was 37.74 (SD = 14.51) years, with an age range from 17 to 63 years, and men with an age range from 16 to 83 (M = 43.56; SD = 16.46) years. Age was divided into three age groups: young adults (≤30 years), middle-aged adults (31–57 years), and older adults (≥58 years). As a result, 56 individuals were classified as young adults (37.3%), 68 as middle-aged adults (45.3%), and 26 as older adults (17.3%). Regarding educational attainment, 37 (24.7%) reported having 9 or fewer years of education, 54 (36%) referred to having 10 to 12 years of education, and 59 (39.3%) an academic degree. Professionally, 41 individuals (27.3%) were associated with the area of enology (winemakers, laboratory analysts, agronomists, cellar store assistants, professors, and university students). For study purposes, subjects were also divided according to their port wine consumption (yes/no). Frequency distribution analysis revealed that 47.3% were port wine consumers. When questioned, a total of 110 (73.3%) individuals reported being willing to buy a port wine-like fragrance.

#### 3.5.2. Descriptive and Univariate Normality Analysis

Table 5 shows the descriptive statistics (range, means, and standard deviations) for the measured variables. The results indicate that the mean age of the sample was 40.73 (SD = 15.76) years, with an age range from 16 to 83 years. Mean values of fragrance classification ranged between 6.55 and 6.65 on a possible scale range of 1 to 9. Absolute values of univariate skewness and kurtosis were within the range of −2 to 2 and were interpreted as normally distributed.

Additionally, to assess whether the product (fragrances) was within the scale considered ideal for the consumer, a JAR scale (from 1 (slightly intense) to 5 (very intense)) was used. Two characteristics were put to the test, aiming to understand if their intensities were in line with what was expected by the consumer, or if otherwise, they would be very or slightly intense. Based on the analysis in Table 6, it is observed that there were differences between the averages of intensity of the odor and color of the fragrances. Moreover, they seemed quite favorable, since consumers considered this feature to be ideally suited to fragrances. Regarding the intensity of the color, it was considered to have a low intensity by most tasters, as it was below the average of the scale (that would be 3). To assess whether there were differences in the fragrance’s preferences regarding the intensity of the odor and color, a MANCOVA (covariate: age) was performed, after validating the assumptions of normality and homogeneity of the variance–covariance matrices. The results point to the absence of significant differences between the preferred fragrances (λ Wilks = 0.98, F_(4,290)_ = 0.93, *p* = 0.45).

It is described in the literature that there are aromas that are associated with specific colors [53]. Authors argue that to facilitate the communication of fragrances or even flavorful fruity beverages, the colors that people tend to associate with certain aromas are used [54]. On the other hand, the presence of color in the fragrance can also be misleading for the detection of an aroma. As an example, Morrot and collaborators [55] observed, during a commented tasting with wine experts, that white wines that were red in color were automatically described as red wines. For this reason, it was decided to present port wine fragrances to consumers, devoid of any color. However, just as when we see a yellow candle, we expect a lemon aroma, perhaps consumers would also expect a characteristic color of port wine for the fragrances, maybe an amber-like color.

#### 3.5.3. Comparative Analysis

A one-way MANCOVA was carried out to compare the effect of gender on the three types of fragrances, after checking the assumptions of normality and homogeneity of variances and covariances matrices (Box’s M = 12.051, F_(6, 157466.478)_ = 1.964, *p* = 0.07). A significant multivariate effect of gender was found after controlling for age (Wilk’s λ = 0.872, F_(3,145)_ = 7.117 *p* < 0.001). One-way analysis of variance showed that only for fragrance 5.1, the differences were significant (*p* < 0.001). Moreover, male scores are higher than females. A possible explanation for our findings might rely on the fact that this fragrance has the highest concentration of benzaldehyde, the aroma of bitter almond being quite evident. In addition to the aromatic compounds giving rise to olfactory sensations, they may, on the other hand, cause sensations of irritation, itching, and/or burning (nasal pungency) [56], which may be the reason why female individuals liked the fragrance less. Moreover, the fact that male individuals scored this fragrance higher may be due to their greater familiarity with a liqueur of bitter almonds, like the Portuguese “Amarguinha”, a sweet alcoholic liqueur made from the seeds of the bitter almond, much like the Italian Amaretto. The results are summarized in Table 7.

To evaluate if the fact of one being a port wine consumer had a statistically significant effect on the classification of fragrances, a one-way MANOVA was carried out, after checking the assumptions of normality and homogeneity of variances and covariances matrices (Box’s M = 6.941, F_(6, 153859.278)_ = 1.131, *p* = 0.341). There was no statistically significant difference between the port wine consumers group on the combined dependent variables (Wilk’s λ = 0.979, F_(3,146)_ = 1.045, *p* = 0.375).

To determine possible differences in fragrance preference by age group, a MANOVA was conducted, after checking the assumptions of normality and homogeneity of variances and covariances matrices (Box’s M = 21.239, F_(12, 29890.356)_ = 1.685, *p* = 0.065). A significant multivariate effect was found (Wilk’s λ = 0.907, F_(6,290)_ = 2.415, *p* = 0.027). A one-way ANOVA produced significant differences in fragrance 5.3 (*p* = 0.023). Post hoc comparisons using Tukey’s reported significant differences between young adults and middle-aged adults (*p* = 0.018). Moreover, young adults scored higher on this fragrance. 

Next, a MANOVA was conducted to compare the effect of educational attainment on the classification of fragrances, indicating no significant multivariate effect (Wilk’s λ = 0.946, F_(6,290)_ = 1.355, *p* = 0.233). Lastly, a MANOVA was conducted to compare the effect of place of residence on the classification of fragrances. The results indicated no significant multivariate effect of residential area (Wilk’s λ = 0.969, F_(3,146)_ = 1.582, *p* = 0.196).

#### 3.5.4. Association between Fragrance Preferences and Sociodemographic Variables

The Chi-square test (χ^2^) of independence was conducted to determine whether there was an association between the type of fragrance and sociodemographic variables gender, age (grouped into three classes), educational attainment, and area of residence. The results indicate that there was no significant association between gender and the type of fragrance (χ^2^_(2)_ = 2.596, *p* = 0.273), educational attainment and the type of fragrance (χ^2^_(4)_ = 4.503, *p* = 0.342), and area of residence and the type of fragrance (χ^2^_(2)_ = 2.241, *p* = 0.326). However, regarding age, a significant association was found (χ^2^_(4)_ = 10.097, *p* = 0.039) (Table 8).

## 4. Conclusions

Because it involves several metabolic processes, the biosynthesis of aromatic compounds, as well as their entire evolution, aroma perception is an extremely complex process. In this way, the idea of wanting to imitate an aroma as enigmatic as that of a tawny port wine was not an easy task. However, there is still no such product on the market, nor a similar study in the literature, valuing this idea for innovation. Besides, the goal was to develop a fragrance that would enhance and celebrate one of the most beloved products of the Portuguese Douro region.

After this work, we can conclude that the sensorial analysis and the panelists were essential for the development of these fragrances. The work started with seven aromatic compounds and six descriptors and ended with only three aromatic compounds and the same descriptors. The last three fragrances developed were those that obtained the highest values of global appreciation by the trained panel.

Classification of the three fragrances, by the final 150 consumers, was positive, obtaining values above the average of the intensity scale. The analysis of the effects of educational attainment and place of residence on the classification of fragrances showed no significant multivariate effects and when testing for an association between fragrance and sociodemographic data (gender, educational attainment, and place of residence), no significant association was found. However, it is important to note that fragrance 5.1, which obtained the highest ratings from male individuals, shared the same preference value as fragrance 5.3. Moreover, fragrance 5.3 was also the one preferred by female individuals.

Regarding the association between fragrance and age, a significant association was found. It should be noted that fragrance 5.3 was the one preferred by young adults, while in the other age groups, the preferences were very balanced.

Concerning the effect of being a port wine consumer, a one-way MANOVA produced no significant differences in the classification of fragrances. However, in the present study, 47.3% of the individuals recruited (32.4% women and 67.6% men) said they were usual consumers of port wine. The usual consumer was understood to consume port wine at least once a week.

Consumers’ opinions are a good quality reference that must be considered. Thus, it would be interesting, in the future, to research which color/colors consumers preferred to be included in fragrances, and, later, to study if this would be a differentiating factor concerning the purchase and use of it. In addition, to study the application of this fragrance in the areas of scent marketing and enogastronomy, now, gourmet restaurants are pairing their feature dishes with recognizable scents and highly appreciated wines.

## Figures and Tables

**Table 1:** Aromatic compounds used and the respective thresholds of perception, for the development of fragrances.

Aromatic Compound	Provider	Detection Threshold
Benzaldehyde	Merck-Schuchardt	1.5–5.5 mg/L (wine) [24]
Furfural	Sigma-Aldrich	0.25–5 mg/L [25]
2-Octanone	Aldrich	1.30 mg/L [26]
Sotolon	Sigma-Aldrich	1–100 µg/L (wine) [27]
Vanillin	IGH Flavours and Technology S.A.	0.2–1.3 mg/L (wine) [28]
α-Ionone	Honeywell Fluka	2.6 µg/L (wine) [29]
β-Damascenone	Aldrich	40–160 µg/L (wine) [30]

**Table 2:** Fragrance samples developed for the first four tasting sessions.

Tasting Session	Fragrance	Concentrations
1st	1.1	Detection threshold
	1.2	2× Detection threshold
	1.3	2× Detection threshold, without sotolon
	1.4	2× Detection threshold, without β-damascenone
	1.5	2× Detection threshold, without α-ionone
	1.6	2× Detection threshold, plus 1× detection threshold of sotolon, β-damascenone, and α-ionone
2nd	2.1	2× Detection threshold
	2.2	2× Detection threshold, without β-damascenone
	2.3	2× Detection threshold, without α-ionone
3rd	3.1	4× Detection threshold
	3.2	4× Detection threshold without β-damascenone
	3.3	4× Detection threshold without α-ionone
	3.4	4× Detection threshold diluted in alcohol obtained from the distillation of a tawny port wine
4th	4.1	4× Detection threshold diluted in the alcohol obtained from the distillation of a tawny port wine
	4.2	10× Detection threshold diluted in the alcohol obtained from the distillation of a tawny port wine
	4.3	Mother solution diluted in the alcohol obtained from the distillation of a tawny port wine

**Table 3:** Fragrance samples (5.1–5.3) developed for the fifth session and respective compound concentration.

Fragrance	Compounds and Concentrations (mg/mL)				
	Benzaldehyde	Vanillin	Sotolon	Furfural	β-Damascenone
5.1	0.240	0.060	0.180	-	-
5.2	0.168	0.096	0.180	0.179	0.114
5.3	0.168	0.096	0.180	-	-

**Table 4:** Descriptors associated with the four wines’ aromas, their frequency of citation, and corresponding aromatic compounds.

Descriptors	Freq. (%)	Descriptors Family	Corresponding Aromatic Compounds and References
Alcohol	100		
“Aguardente” or Brandy		Alcohol	Ethanol
Raisins	100		
Plum			
Peach			
Dry Fig			eugenol [34,35,36]
Roasted Chestnuts		Dry Fruits	benzaldehyde [34,37,38]
Peanut			
Hazelnut			
Nut			
Almond			
Cinnamon	80		
Cumin			guaiacol [34,35,38]
Clove		Spices	vanillin [34,35,38,39]
Vanilla			β-cyclocitral [40]
Nutmeg			
Wood	76	Wood	furfural [34,35,37,38,39] 2-octanone [34,35,37,38,39] sotolon [34,35,38,41]
Sweet/Honey	65	Sweet/Honey	phenylacetaldehyde [37]
Floral/ Dry Flowers	45	Floral/ Dry Flowers	β-damascenone [34,35,37,40] α-ionone [34,35,37,38,40]
Caramel	35		
Chocolate	28		
Burnt/Smoke	15		
Acid/Vinegar	10	Others	
Old/Mold	5		
Gooseberry	5		

**Table 5:** Descriptive and univariate normality analysis.

Variables	Range	M	SD	Skewness	Kurtosis
Age	16–83	40.73	15.759	0.272	−0.803
Fragrance 5.1	4–8	6.63	1.132	−0.516	−0.696
Fragrance 5.2	2–9	6.55	1.190	−0.794	1.294
Fragrance 5.3	3–9	6.65	1.362	−0.742	0.199

**Table 6:** Characterization of the preferred fragrances concerning the intensity of the odor and the color.

	Fragrance	M ± SD	N
**Odor intensity**	5.1	3.16 ± 0.65	44
	5.2	3.30 ± 0.88	44
	5.3	3.11 ± 0.45	62
**Color intensity**	5.1	2.36 ± 0.84	44
	5.2	2.25 ± 0.89	44
	5.3	2.39 ± 0.91	62

**Table 7:** Means (M), standard deviation (SD), and univariate effects of the fragrances by gender.

	Female (M ± SD)	Male (M ± SD)	F	*p*
Fragrance 5.1	6.21 ± 1.201	7.03 ± 0.903	22.510	<0.001
Fragrance 5.2	6.42 ± 1.235	6.68 ± 1.141	1.670	0.198
Fragrance 5.3	6.55 ± 1.385	6.74 ± 1.342	0.746	0.389

**Table 8:** Association between age and fragrance.

Age	Frag. 5.1		Frag. 5.2		Frag. 5.3			
	n	Exp	n	Exp	n	Exp	*p*	v
≤30	10	16.4	14	16.4	32	23.1	0.039	0.259
31–57	25	19.9	22	19.9	21	28.2		
≥58	9	7.6	8	7.6	9	10.7		

## Supplementary Materials

The following are available online at https://www.mdpi.com/2304-8158/9/9/1244/s1. Supplementary Form 1S: Recruitment Form, Supplementary Form 2S: Vocabulary Generation Form, Supplementary Form 3S: Tasting Form 1, Supplementary Form 4S: Tasting Form 2, Supplementary Form 5S: Tasting form 3, Supplementary Form 6S: Test Sheet JAR (Just About Right).
