# Supplementary material for: Creation and Acceptability of a Fragrance with a Characteristic Tawny Port Wine-Like Aroma
Source: Foods. 2020 Sep 6;9(9):1244. doi: 10.3390/foods9091244 (PMC7555520; doi:10.3390/foods9091244)
Supplement: Supplementary file 1 [file foods-09-01244-s001.zip › Supplementary form 1S.docx]

**Recruitment Form**

Name**: ________________________________________** Date**: _______**

Sex**: _______** Age**:** **_______** Career**: _______**

1. What is your interest and motivation in participating in sensory analysis tests?

**____________________________________________________________**

1. Do you have a health problem that affects your senses?

**____________________________________________________________**

1. Do you have any intolerance, aversion, or allergy to a food/ingredient?

**____________________________________________________________**

1. What is your availability for the sensory analysis tests?

**____________________________________________________________**

1. Is wine part of your eating habits?

**____________________________________________________________**
