# Supplementary material for: Creation and Acceptability of a Fragrance with a Characteristic Tawny Port Wine-Like Aroma
Source: Foods. 2020 Sep 6;9(9):1244. doi: 10.3390/foods9091244 (PMC7555520; doi:10.3390/foods9091244)
Supplement: Supplementary file 1 [file foods-09-01244-s001.zip › Supplementary form 2S.docx]

**Vocabulary Generation Form**

Name**: ________________________________________** Date**: _______**

Describe the sensations/perceptions suggested by the presented Port Wine according to the indicated property, using vocabulary that is familiar to you.

**Smell**

|  |
| --- |
