# Supplementary material for: Creation and Acceptability of a Fragrance with a Characteristic Tawny Port Wine-Like Aroma
Source: Foods. 2020 Sep 6;9(9):1244. doi: 10.3390/foods9091244 (PMC7555520; doi:10.3390/foods9091244)
Supplement: Supplementary file 1 [file foods-09-01244-s001.zip › Supplementary form 3S.docx]

**Tasting Form 1**

Name: **_________________________** Date: **________** Sample Code

**Smell**

|  | | **Little intense** | | | **Medium** | | | **Very intense** | | |
| --- | --- | --- | --- | --- | --- | --- | --- | --- | --- | --- |
| **Overall intensity** | | **1** | **2** | **3** | **4** | **5** | **6** | **7** | **8** | **9** |
| **Alcohol** |  |  |  |  |  |  |  |  |  |  |
| **Wood** |  |  |  |  |  |  |  |  |  |  |
| **Spices** |  |  |  |  |  |  |  |  |  |  |
| **Sweet/Honey** |  |  |  |  |  |  |  |  |  |  |
| **Floral/Violets** |  |  |  |  |  |  |  |  |  |  |
| **Dry fruits** |  |  |  |  |  |  |  |  |  |  |

**Global Appreciation**

| **Unpleasant** | | | **Medium** | | | **Pleasant** | | |
| --- | --- | --- | --- | --- | --- | --- | --- | --- |
| 1 | 2 | 3 | 4 | 5 | 6 | 7 | 8 | 9 |

Comments:

Thank you!
