# Supplementary material for: Creation and Acceptability of a Fragrance with a Characteristic Tawny Port Wine-Like Aroma
Source: Foods. 2020 Sep 6;9(9):1244. doi: 10.3390/foods9091244 (PMC7555520; doi:10.3390/foods9091244)
Supplement: Supplementary file 1 [file foods-09-01244-s001.zip › Supplementary form 5S.docx]

**Tasting Form 3**

Name: **_____________________** Date: **________** Gender (F/M): **______** Age: **_____**

You are presented with 3 samples of different fragrances. Try Them.

Do you find fragrances like a Port Wine aroma? Yes ____ No ____

Write which sample you like more and why.

Thank you!
