# Supplementary material for: Creation and Acceptability of a Fragrance with a Characteristic Tawny Port Wine-Like Aroma
Source: Foods. 2020 Sep 6;9(9):1244. doi: 10.3390/foods9091244 (PMC7555520; doi:10.3390/foods9091244)
Supplement: Supplementary file 1 [file foods-09-01244-s001.zip › Supplementary form 6S.docx]

**Test Sheet JAR (Just About Right)**

Name: **_____________________** Date: **________** Gender (F/M): **______** Age: **_____**

Career: **_____________________** Nationality: **_____________________**

To raise and keep alive the tradition of what the best is done in the Douro region and, in Portugal, a fragrance of Port wine was created.

Are you a regular consumer of Port Wine? Yes ____ No ____

**You are presented with 3 fragrance samples.**

How would you, generally, rate the fragrance number 1?

| Love | Really like | Like | Like it lightly | Don't like or dislike | Don't like it very much | Don't like | Don't like at all | Hate |
| --- | --- | --- | --- | --- | --- | --- | --- | --- |
|  |  |  |  |  |  |  |  |  |

How would you, generally, rate the fragrance number 2?

| Love | Really like | Like | Like it lightly | Don't like or dislike | Don't like it very much | Don't like | Don't like at all | Hate |
| --- | --- | --- | --- | --- | --- | --- | --- | --- |
|  |  |  |  |  |  |  |  |  |

How would you, generally, rate the fragrance number 3?

| Love | Really like | Like | Like it lightly | Don't like or dislike | Don't like it very much | Don't like | Don't like at all | Hate |
| --- | --- | --- | --- | --- | --- | --- | --- | --- |
|  |  |  |  |  |  |  |  |  |

**Consider your favorite fragrance:**

Did you buy the product (repeat purchase)? Yes ____ No ____

Would you use the fragrance in your home or for another purpose? Yes ____ No ____

What:

Consider the typical Port wine aroma. Indicate whether you think the **characteristic smell** is less or more intense than what you consider to be ideal for you.

| Very little intense | Little intense | Ideal | Intense | Very intense |
| --- | --- | --- | --- | --- |
|  |  |  |  |  |

Consider what the color of a fragrance would look like. Indicate whether you think the **characteristic color** is less or more intense than what you consider to be ideal for you.

| Very little intense | Little intense | Ideal | Intense | Very intense |
| --- | --- | --- | --- | --- |
|  |  |  |  |  |

Thank you!
